# Challenging Growing Regional Inequality: Area-Based Policies in Germany as a Path to Equivalent Living Standards?

**DOI:** 10.1007/s11615-025-00636-4

**Published:** 2025-10-15

**Authors:** Naomi Alcaide Manthey

**Affiliations:** Department of Geosciences and Natural Resource Management, University of Copenhagen, Copenhagen, Denmark

**Keywords:** Disadvantaged areas, Regional inequality, Polarization, Public service provision, Citizen engagement, Regionale Strukturförderung, Regionale Ungleichheit, Gleichwertige Lebensverhältnisse, Polarisierung, Daseinsvorsorge

## Abstract

In recent years, inequality has deepened across many European countries. Targeted investments have seen limited success, and many democracies face growing polarization as disparities along economic, demographic, and social lines persist. In Germany, residents of structurally disadvantaged areas increasingly feel overlooked by policies focused on economic growth rather than everyday needs. This article explores what residents in these regions perceive as lacking in their living environments, what influences their satisfaction or dissatisfaction, and how well current policy, specifically the funding scheme of the Joint Task for the Improvement of Regional Economic Structures (GRW), responds to these concerns. Based on fieldwork in three GRW-eligible communities, the study compares local perceptions with the scheme’s design and recent reforms. While reforms show a shift toward basic infrastructure, important aspects of daily life, such as transport, social spaces, and local retail, remain difficult to support under current funding rules. Civil society actors face limited access to resources, and municipalities often lack the capacity to apply for and implement available funds. These findings point to the need for better alignment between funding mechanisms and the lived realities in disadvantaged regions.

**Supplementary Information:**

The online version of this article (10.1007/s11615-025-00636-4) contains supplementary material, which is available to authorized users.

## Abstract (de)

In den letzten Jahren hat sich die Ungleichheit in vielen europäischen Ländern weiter verschärft. Gezielte Investitionen waren bislang nur begrenzt erfolgreich, und viele Demokratien sehen sich mit wachsender Polarisierung konfrontiert, da wirtschaftliche, demografische und soziale Unterschiede fortbestehen. In Deutschland fühlen sich Bewohnerinnen und Bewohner strukturschwacher Regionen zunehmend von einer Politik übergangen, die sich vorrangig auf Wirtschaftswachstum statt auf alltägliche Bedürfnisse konzentriert. Dieser Artikel untersucht, welche Mängel sie in ihrem unmittelbaren Umfeld wahrnehmen, welche Faktoren ihre Zufriedenheit oder Unzufriedenheit beeinflussen und inwiefern aktuelle Förderprogramme wie die GRW-Förderung auf diese Anliegen eingehen. Auf Grundlage von Feldforschung in drei GRW-förderfähigen Gemeinden vergleicht die Studie lokale Wahrnehmungen mit der Ausgestaltung des Programms und den jüngsten Reformen. Diese zeigen zwar eine stärkere Ausrichtung auf Basisinfrastruktur, doch zentrale Aspekte des Alltags – wie Verkehrsanbindung, soziale Begegnungsräume und der lokale Einzelhandel – bleiben nach den geltenden Förderregeln weiterhin schwer zu unterstützen. Zivilgesellschaftliche Akteure haben nur eingeschränkten Zugang zu Ressourcen, und viele Kommunen verfügen nicht über die nötigen Kapazitäten, um die verfügbaren Mittel effektiv zu beantragen und einzusetzen. Die Ergebnisse unterstreichen die Notwendigkeit, Fördermechanismen besser mit den Lebensrealitäten in benachteiligten Regionen abzustimmen.

## Introduction

Area-based policies have been criticized and just as whole-heartedly defended by scholars who emphasize the fact that in times of limited resources, area-based policies allow the concentration of funds in areas where disadvantages accumulate. Aside from other reasons, this is why, in the field of regional development and structural support to disadvantaged areas, area-based policies are the general rule for resource allocation. Even though the general approach of targeting areas having certain characteristics is rarely contested these days, the selection criteria and the funds that get allocated through such policies certainly are subjects of discussions. Contributing to this discussion, this article will examine how, in the case of Germany, large funding schemes interact with the lived realities, needs, and priorities of citizens in disadvantaged regions. Though attention will be paid to recent reforms and the ways in which these reforms allow for individual solutions in targeted regions, the study adopts an exploratory approach based on three case studies, rather than evaluating public funding instruments against a fixed set of assessment criteria.

This focus becomes particularly relevant in light of current broader sociopolitical developments. The issue of growing disparities and interregional inequality has continued to gain attention in recent years as political polarization has increased and seemed to intensify along the lines of socioeconomic inequality. Despite a general rise in right-wing voters, a particular success of populist parties can be observed in areas with high levels of place resentment and sociodemographic decline, two factors that are closely connected (Arzheimer and Bernemann 2024, p. 182). Though demographic decline cannot be traced back solely to economic circumstances, it certainly does create a downward spiral affecting infrastructure, services, quality of life, and, ultimately again, economic activity in a region, which makes it a cause of political discontent and ethnocentrism (Salomo 2019). Nevertheless, this web of causes contributing to decline and regional inequality consists of more than just economic factors, even if area-based policies and development aid historically tend to focus on solely economic measures of support and success.

The inception of area-based policies and partnerships as a standard policy tool in Europe is commonly associated with the rise of growing inequality in Europe during the 1980s and 1990s (Atkinson and Zimmermann 2018) following the globalization of economic development. Haase and McKeown (2003) describe how these instruments were often applied in a “context of crisis situations” (p. 2) and frequently led to disillusions due to the resilience of inequality and poverty toward public interventions. In response to unequal spatial distribution of socioeconomic resources, area-based policies target areas affected by economic, social, and/or infrastructural disadvantage through the allocation of funding (i.e., Wardenburg and Brenner 2020). Building on this approach, Andersson and Musterd (2005) outline three rationales for the widespread adoption of area-based policies, either as alternatives or complements to national strategies:

1. Social problems are unevenly spatially distributed. Areas of concentrations allow the addressing of a large number of people in disadvantaged positions in a cost-effective manner.
2. Concentration of poverty poses a problem for democratic welfare states because it makes inequality more visible. Ethnic and racial components can indicate failing integration policy.
3. Negative neighborhood effects are feared to create disadvantage for formerly not disadvantaged individuals living in the deprived area.

These rationales - namely, addressing spatial concentrations of the disadvantaged, mitigating the visibility and political implications of poverty in democratic welfare states, and preventing negative neighborhood effects - help explain why area-based policies have gained such widespread acceptance (Barca 2009; Atkinson and Zimmermann 2018) even as critiques of their effectiveness continue to emerge. Critics argue that many designated areas are “artificial constructs, often unrelated to the causes of the problems being addressed, containing disparate populations with little in common” (Atkinson and Zimmermann 2018, p. 29). There is also concern that defining specific characteristics for area selection can lead to the exclusion of other disadvantaged groups (Andersson and Musterd 2005). Some suggest that antipoverty measures should instead be based on criteria such as social welfare payments, income tax codes, and entitlements to free state services (Haase and McKeown 2003).

Atkinson and Zimmermann (2018) analyze the shortcomings of traditional area-based policies and describe the characteristics of more recent area-based interventions and the emergence of place-based policies, which seek to avoid target-area definition along administrative lines and instead attempt to base area designation based on territorial, social, and economic factors. Referring to the Barca (2009) commissioned by the European Commission, they also describe the need for systemic change, one that includes more citizen involvement and cooperation in the process of resource distribution in order to account for local needs and preferences. In line with community development theory, this demand pays tribute to the fact that top-down funding schemes often focus exclusively on economic criteria and interventions, which can misalign with citizens’ needs in funding-eligible areas. Kretzmann and McKnight (1993) underline the need for local citizens to not only be selectively involved in formalized development participation processes but to be considered leaders of development that is to be supported by local and national governments through capacity-building resources. McKnight and Kretzmann are considered foundational for the asset-based development approach, which emphasizes the need to move away from classic deficit-focused policy approaches toward an asset-oriented focus that fosters community strength and follows the citizens’ lead in addressing needs for improvements.

General tendencies of urban and regional development policies have shown the acknowledgement of this line of thinking by introducing corresponding funding schemes that focus on collaborative development measures. Nevertheless, large-scale area-based funding programs targeted at the alleviation of inter- and intraregional inequality mostly focus on economic indicators and measures, rather than community-centered measures. Despite advances through reforms, which will be examined more closely in the following section, a gap remains between collaborative rhetoric and an economic focus of area-based funds. To unpack this tension, three case studies from Germany will be analyzed, guided by the following core questions:

- What deficits do residents in structurally disadvantaged areas identify in their living environment, and which social and infrastructural factors drive their satisfaction or dissatisfaction?
- How well do existing funding schemes address identified deficits, and where do gaps remain?

To answer these questions, the instruments available to address potential deficits and improve local living standards will be examined. The following section thus provides a review of major German funding programs and their eligibility rules and reform trajectories, which will later be used to compare them with findings from the three case studies.

## Exploring Policy: Equality, Equity, or Equivalence—How to Set Goals in Combatting Regional Inequality?

In Germany, basic law formulates the legislative competence and duty of the federal government to take action in achieving “equivalent living conditions.”^1^ Due to the unique history of Germany, equality and unity on territorial, economic, political, and social grounds have been a particular challenge. Ever since the German reunification in 1990, equalizing living standards in both the former East Germany and the former West Germany has been on all recent governments’ agendas. Despite such claims, inequality in Germany is not only manifested along territorial lines of the former inner-country border, but inequality of living standards is also growing between geographical units: urban and rural spaces, inner-city and suburban areas, former industrial and nonindustrial cities, growing and shrinking spaces (Weck and Beißwenger 2014; Wardenburg and Brenner 2020).

The occurrence of shrinkage has had especially wide-reaching effects on demographic and economic resources of the respective areas: People leave and leave behind a built environment that was designed to accommodate larger populations than what they are left with. The consequence is an underutilization of infrastructures, loss of tax money and public budgets, departure of local companies, and growing unemployment (Lang and Tenz 2003; Bartholomae and Schoenberg 2017). Billions of euros have been invested to both stop this development and mitigate consequential damage.

Despite those sums being spent, the public satisfaction with the effects of the investments is moderate at best. This could lead to a general questioning of the efficiency of area-based policies. Other arguments, however, show the reasons for why the improvement of living conditions can and maybe should still be pursued through area-based approaches. Lupton and Tunstall (2003) list five rationales for area-based policies, which include, most importantly, the observation that there often is a spatial concentration of poverty in certain areas, which “may have cumulative and qualitatively different effects on individuals, organisations, and infrastructure than less concentrated poverty” (p. 4). In contemporary Germany, this assumption can, besides the obvious economic effects, also be translated into a spatial accumulation of insufficient access to infrastructure.

The concept of social exclusion comes into play when a territorial accumulation of poor access to infrastructure is paired with low socioeconomic status. Lupton and Tunstall describe the risk of social exclusion in the context of area-based policy as follows (2003, p. 11):

“(…)elements that go beyond material deprivation and poverty, such as lack of social participation, interaction and the agency of the excluded and the excluders, and the role of subjective attitudes. Social exclusion is concerned with process, and may develop if poverty or disadvantage are sustained over time. All of these elements make social exclusion difficult to define and measure.”

Whether places with poor economic opportunities and poor access to infrastructures are called disadvantaged, structurally weak, peripheral, left-behind, or places that don’t matter, they all refer to communities facing multiple challenges, which provide little cause for optimism about their future. In their work on left-behind places, John Tomaney and his colleagues (Tomaney et al. 2024) emphasize the emotional challenges when, in addition to economic and industrial restructuring, the closures of social infrastructures such as pubs, youth clubs, and high streets further challenge local social identity and morale. The fact that such occurrences correlate with increasing levels of support for populist parties underlines the importance of addressing not only economic but also identity-forming social infrastructures (Rodríguez-Pose 2018; Tomaney et al. 2024). These global tendencies can also be found in Germany; in particular, authors focused on the former East Germany have emphasized that focusing solely on economic measures such as unemployment rates, which in comparison to other European countries such as France are not worryingly high, leads to a misunderstanding of the relative deprivation and anxiety shared by many residents in eastern German regions (Salomo 2021, p. 204).

In light of tight financial budgets, relying on public institutions to maintain and provide all types of (social) infrastructures and services is both financially and administratively unfeasible. So far, there is no system in place that helps prioritize which infrastructures are crucial for both basic supply and the protection of place identities in disadvantaged areas. On the contrary, as of today there is no formal minimum standard for infrastructure, especially social infrastructure (Danielzyk 2014; Winkel 2018), which could be used to evaluate “regional attractiveness” or “local disadvantages” and could further inform guidelines or minimum standards to be used in public funding schemes.

Rather than a unified “catalog” of what minimum basic infrastructure or living standards should entail, a central approach toward the achievement of the constitutionally established goal of equivalent living conditions has for a long time been an adaptation of the central places theory, which constitutes that relevant infrastructures and services are to be provided in central places that are to be reached for all citizens within a certain radius. Whereas Christaller (1968) based his theory on historically existing settlement and function patterns in southern Germany, regional planning has tried to adapt the theory to active planning in Germany. Subsequent research, however, found that the system identified by Christaller in southern Germany could not be applied to states (dt. Länder) such as Thüringen (Behrens 2018). Nevertheless, Christaller’s theory had formed the foundation for an early regional funding scheme in the 1950s called the “Central Place Program,” and today most states in Germany have state-specific central place catalogs, which are not binding but refer to distances between settlements, infrastructure, and service providers within the region.

In relation to area-based funding schemes, it is to be noted that there is a proven correlation between municipal debt and household debt (Rösel 2016), which illustrates the system of dependency that exists in relation to economic and demographic change. Another challenge that arises with the implementation of the central places concept is the fiscal asymmetry that exists when citizens use infrastructure and services in central places but pay taxes elsewhere, a problem similar to the financial imbalance that arises when an increasing number of residents become dependent on public services and social welfare (e.g., due to aging, unemployment, or illness), whereas the municipal income through taxes decreases (Becker and Naumann 2020).

This leads back to the issue of determining which basic services and infrastructures are to be guaranteed by the (national) state and which responsibilities can be passed on to local governments and municipalities, which then have to align the level of infrastructure and service provision to their available budget. This discrepancy alone underlines the fact that the principle of “equivalence of living standards” can never refer to a truly “comprehensive and equal provision, but that differences, i.e. between rural and densely populated areas, can remain or increase as long as a certain minimum offer is guaranteed” (Küpper and Mettenberger 2020, p. 23). The fact that neither politicians nor scientists nor civil society organizations have so far come up with a unified list of criteria of what is included in a minimum setup of basic infrastructures and services illustrates the heterogeneity of needs in different parts of the country. There is a discrepancy between subjective needs of citizens and objective supply data, since, depending on the geographical location, citizens expect different levels of accessibility and, depending on individual resources, they are more or less capable of reaching infrastructures outside of their immediate living environment (Steinführer and Küpper 2013; Küpper and Mettenberger 2020).

Former German President Horst Köhler appeared rather critical toward the applicability of “equivalent living standards” and compared the implementation of equivalence with the establishment of a subsidy state (Eubel 2004), a statement that received strong criticism, especially from politicians in the former East German states. But he was not alone with his doubt of the feasibility of equivalent living standards. Years after his statement, the debate on this continues to divide policy and planners alike. Some voices agreed with the assumption that it would be unfeasible to implement equivalent living standards in such a heterogeneous geographical landscape as Germany and called for alternative solutions, such as “self-responsibility spaces,” in which civil society is a firmly planned and integrated provider of infrastructure supply (Aring 2013). Others emphasized the risk of expecting too much of the civil society in compensating for public deficiencies and highlighted the risk for further burdens on social cohesion and the resulting consequence of a growing polarization in the country (Franz et al. 2018; Salomo 2019, 2021; Becker and Naumann 2020).

While the transfer of responsibilities toward civil society stakeholders has not been implemented in a formal process or policy, over recent decades many network structures have formed that are crucial for the maintenance of many institutions and services in financially struggling communities. Within these networks, municipalities take over many responsibilities, but always alongside welfare institutions, churches, self-help organizations, and other civil society organizations, which increasingly take over responsibilities related to social infrastructures (Winkel 2018; Alcaide Manthey 2024).

**Fig. 1:**
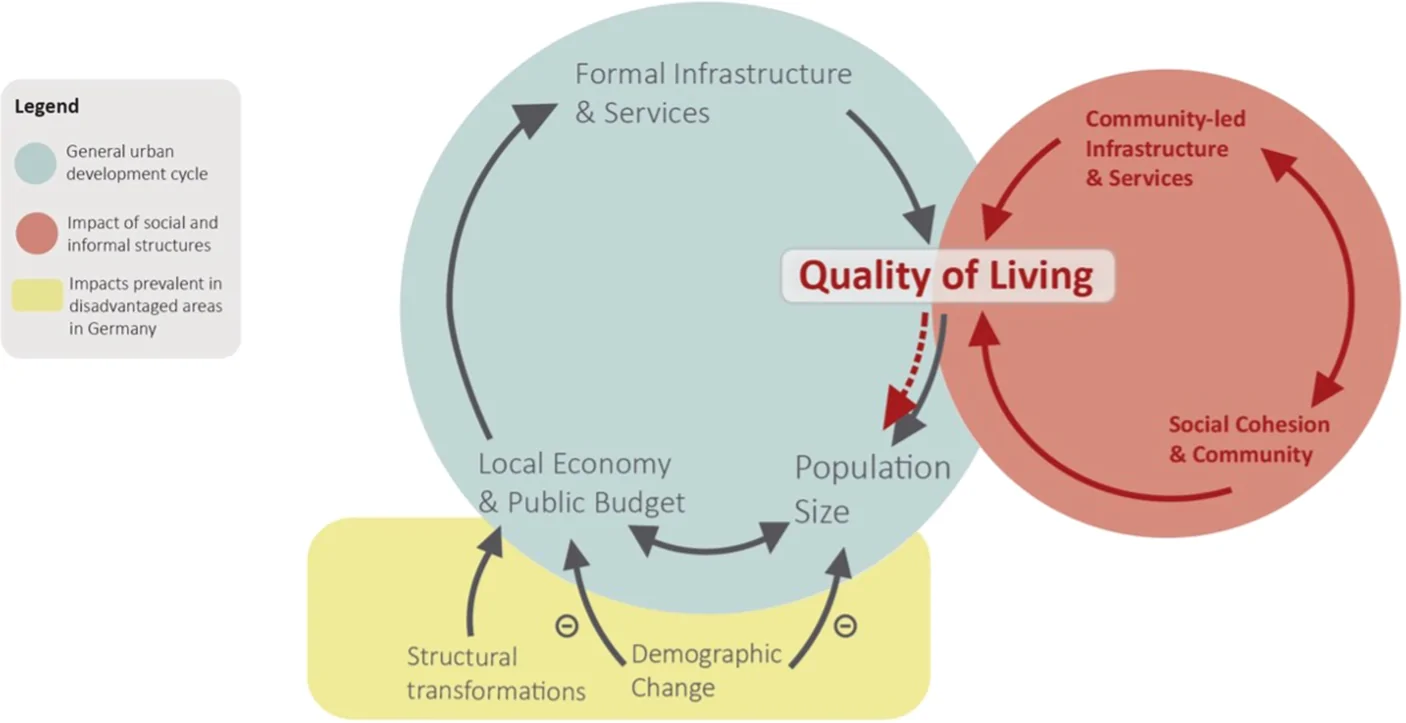
Urban development cycle in disadvantaged areas and the impact of community strength. A large circular cycle illustrates the interdependence of formal infrastructure and services, the local population size, and the local economy and public budget. The latter two are particularly negatively affected in structurally disadvantaged areas through the effects of demographic change and structural transformations. A second cycle intersects and represents the potential impact of social and informal community structures, such as community-led infrastructure and services and social cohesion and community, which can increase the local quality of living despite negative externalities in disadvantaged areas. Source: Alcaide Manthey 2024

Research has shown the potential that lies in those nongovernmental stakeholders and the dependency on them to interrupt downward spirals of regional decline, as is illustrated in Fig. 1:

The illustration shows how the local community can impact the quality of living in disadvantaged areas and - with the right support provided through public institutions - can put a stop to the downward spiral that many shrinking and disadvantaged areas are facing. In order to minimize the risk of overburdening civil society and other nongovernmental stakeholders, public and especially municipal resources need to be put to use accordingly. Local communities provide an irreplaceable resource but cannot compensate for the role of public institutions in carrying the responsibility for local living standards (Tautz et al. 2018; Alcaide Manthey 2024).

The Federal Funding System for Structural Development Regions (*Gesamtdeutsches Fördersystem für strukturschwache Regionen*; GFS) is institutionally in charge of this responsibility, which has been the result of a comprehensive reform of previous funding schemes. Since 2020, this reform has unified about 20 different regional funding schemes under one roof. Investments are being channeled toward areas in demand following the categorization of the Joint Task for the Improvement of Regional Economic Structures (GRW). Because the GRW funding scheme is the largest program addressing regional inequality, a subsequent analysis will further focus on its impact and its reform in recent years.

The GRW funding as a regional economic development policy was initiated in 1969 with the primary objective of promoting equivalent living conditions across regions within the former West Germany, the Federal Republic of Germany. It is closely aligned with the European Regional Development Fund and focuses explicitly on the stimulation of economic growth and employment. The GRW aims to reduce regional disparities and promote development in line with European Union (EU) cohesion goals and is closely coordinated with EU regional policy, often leveraging cofinancing from the European Regional Development Fund to amplify its impact. The framework for GRW is agreed upon by federal and state governments, ensuring that national support aligns with EU priorities and is subject to EU state aid and competition law, which restricts member states from issuing national funding schemes that could distort competition. The eligibility of regions and aid intensities under GRW must therefore comply with EU regional aid guidelines and requires approval from the European Commission (Alecke and Mitze 2023; BMWK 2024b).

The funding program is divided into two main schemes: First, it subsidizes industrial investments of firms, and second, it subsidizes municipalities to enhance economically relevant infrastructures, such as those related to transportation or communication.

The GRW funding areas are determined by four indicators: regional productivity (gross domestic product per employed person), average unemployment rate, development of the number of people of working age (2017 to 2040), and - since the latest reforms - an infrastructure indicator. Based on those criteria, 77.5% of the funds were allocated to former East German regions between 2000 and 2014, in an area representing 15% of the total German population (Wardenburg and Brenner 2020). The GRW is the single largest and most important regional policy tool, with the main objective of creating equivalent living standards in all regions of Germany (BMWK 2022a). Despite the program’s considerable financial investment, critics emphasize the fact that sustained long-term economic improvements have not translated into enhanced regional equivalence regarding the quality of life within these areas (Wardenburg and Brenner 2020).

**Fig. 2:**
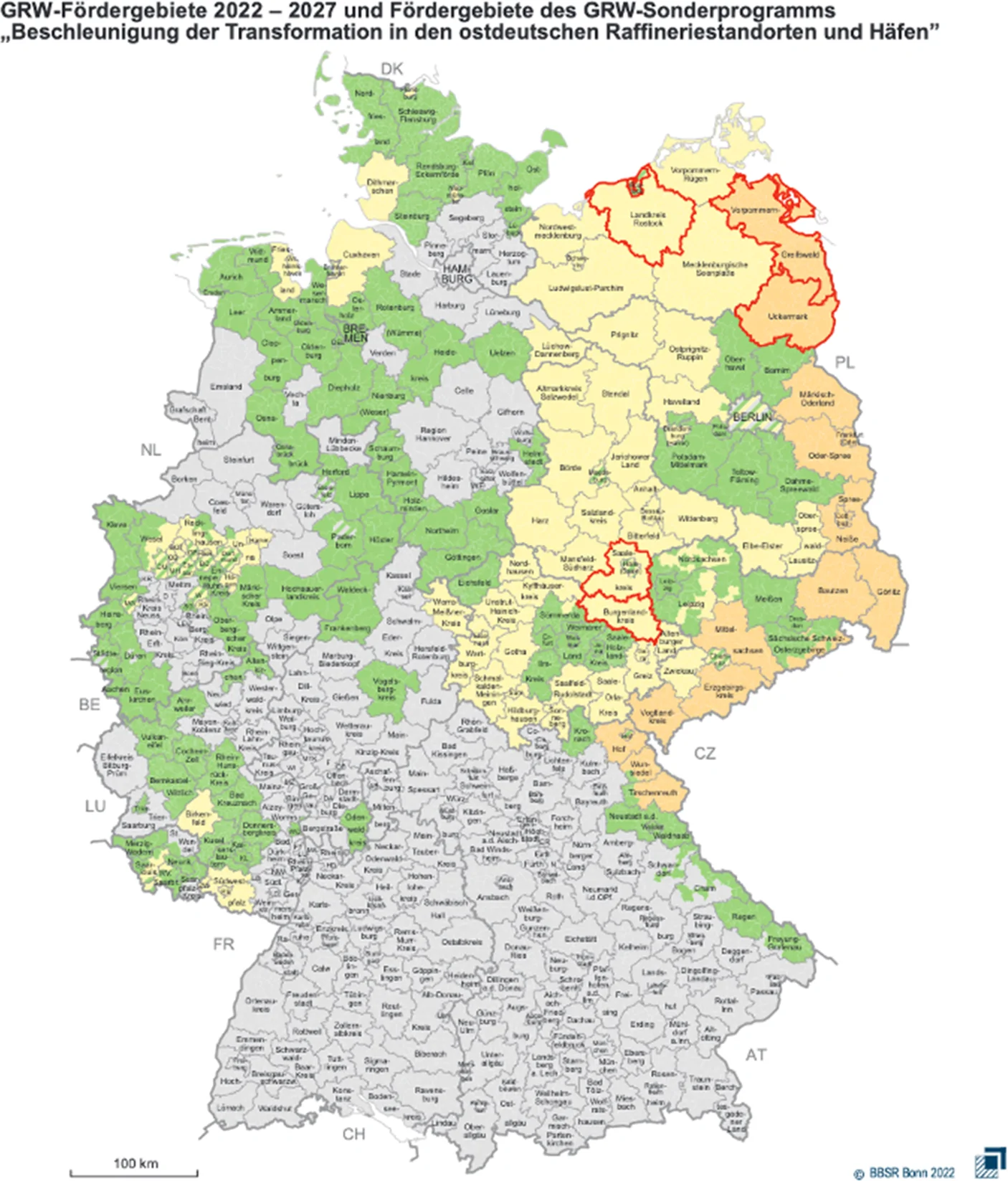
Areas eligible for the Joint Task “Improving the regional economic structure” in the period 2022–2027 and the Joint Task for the Improvement of Regional Economic Structures (GRW) special program “Accelerating the transformation in the East German refinery sites and ports.” The figure depicts a map of Germany, illustrating the current distribution of GRW funding zones. Colored areas cover all areas of the former East Germany and scattered areas throughout the north and west of the country. No funding zones are located in the two southernmost regions of Germany except for some funding areas along the Czech Republic’s southwest border. Source: BMWK, 2024b

Though the 2020 evaluation of the GRW funding program draws a generally positive conclusion regarding its overarching objective to protect and create long-term employment opportunities (BMWK 2020), the continuing objective of ensuring equivalent living standards throughout all regions was further emphasized by the newly founded commission on equivalent living standards (dt. *Kommission Gleichwertige Lebensverhältnisse*) in July 2019. Whereas for a long time after the German reunification these structural funds have been available only to regions that were formerly in East Germany, in 2020 the guidelines were adapted to include all structurally disadvantaged areas in Germany along with further reforms of the program, which will be discussed in more detail later in this paper. Based on a set of criteria that can qualify a region for funding targeted at improvements of economic activity and structural investments, the geographical distribution for the funding period of 2022–2027 areas appears as depicted in Fig. 2.

The green areas indicate the category for the lowest levels of funding allocated to them, with yellow areas marking higher funding needs and orange areas additionally qualifying for extra funding due to specifics of border locations. The grey areas are nonfunding zones for this specific scheme, and the areas bordered in red are specifically assigned areas for a special program for refinery sites and ports.

The funds that are distributed by the respective states are targeted at securing and creating employment and income; increasing growth and prosperity; offsetting locational disadvantages; accelerating transformation processes toward a climate-neutral and sustainable economy (BMWK 2024b: 5). The GRW funding is the largest funding source for regional development in Germany and seeks to achieve those goals through the medium- to long-term strategy of accelerating regional economic activity and strengthening infrastructure that contributes to commercial attractiveness. Although infrastructure that improves local basic services and therefore increases a location’s attractiveness for businesses can be eligible for funding, three-quarters of all funds during recent evaluation periods have been used for commercial economic investments and only one-quarter for infrastructural investments (BMWK 2020, p. 9). This contradicts the overarching goal of equivalent living conditions, as a growing body of literature highlights that citizens’ perceptions of good living conditions in disadvantaged areas extend well beyond purely economic factors (cf. Cooke et al. 2016; Mäki-Opas et al. 2022). Browne-Yung et al. (2016) demonstrate that for low-income residents, aspects such as neighborhood safety, the absence of disorder, the quality of the built environment, and the reputation of the area are central to well-being. Their research, based on interviews and surveys, shows that social capital, community engagement, and access to clean, well-maintained public spaces significantly shape residents’ satisfaction and sense of security. These findings underscore the importance of considering the social and environmental context when assessing quality of life in disadvantaged regions.

As influential regional policy experts within Europe, Barca et al. (2012) further argue that effective policy must be place-based, reflecting the specific needs and assets of local communities. They emphasize that interventions should address social cohesion, institutional quality, and lived experiences, not just economic indicators, in order to improve the well-being of residents in disadvantaged areas. Complementing this perspective, Mäki-Opas et al. (2022) use the capability approach to assess quality of life in disadvantaged groups, showing that noneconomic factors - such as autonomy, social participation, and supportive environments - are as crucial as material resources for well-being. They find that education and income affect quality of life mainly by shaping individuals’ capabilities and opportunities. This suggests that policies should focus on expanding people’s choices, opportunities, and social inclusion, not just improving economic conditions. This is further supported by an influential review of widely validated academic instruments and common policy tools for well-being assessment, such as the World Health Organization Quality of Life - Brief Version (WHOQOL-BREF), which Cooke et al. published in 2016 (Cooke et al. 2016). They emphasize the need to integrate both subjective perceptions and objective living conditions, as only a multidimensional view of well-being can help create circumstances that improve conditions for diverse populations and settings. Aspects related to environmental conditions include safety, home, finances, services, information, leisure, environment, and transport, and individual subcategories from the other groups include, e.g., positive feelings, esteem, relationships, and social support (Harper et al. 1998), some of which are based on very individual perceptions but are strongly impacted by the living environment of individuals.

### Effectiveness of Area-Based Policies in Addressing Regional Inequality in Germany

**Fig. 3:**
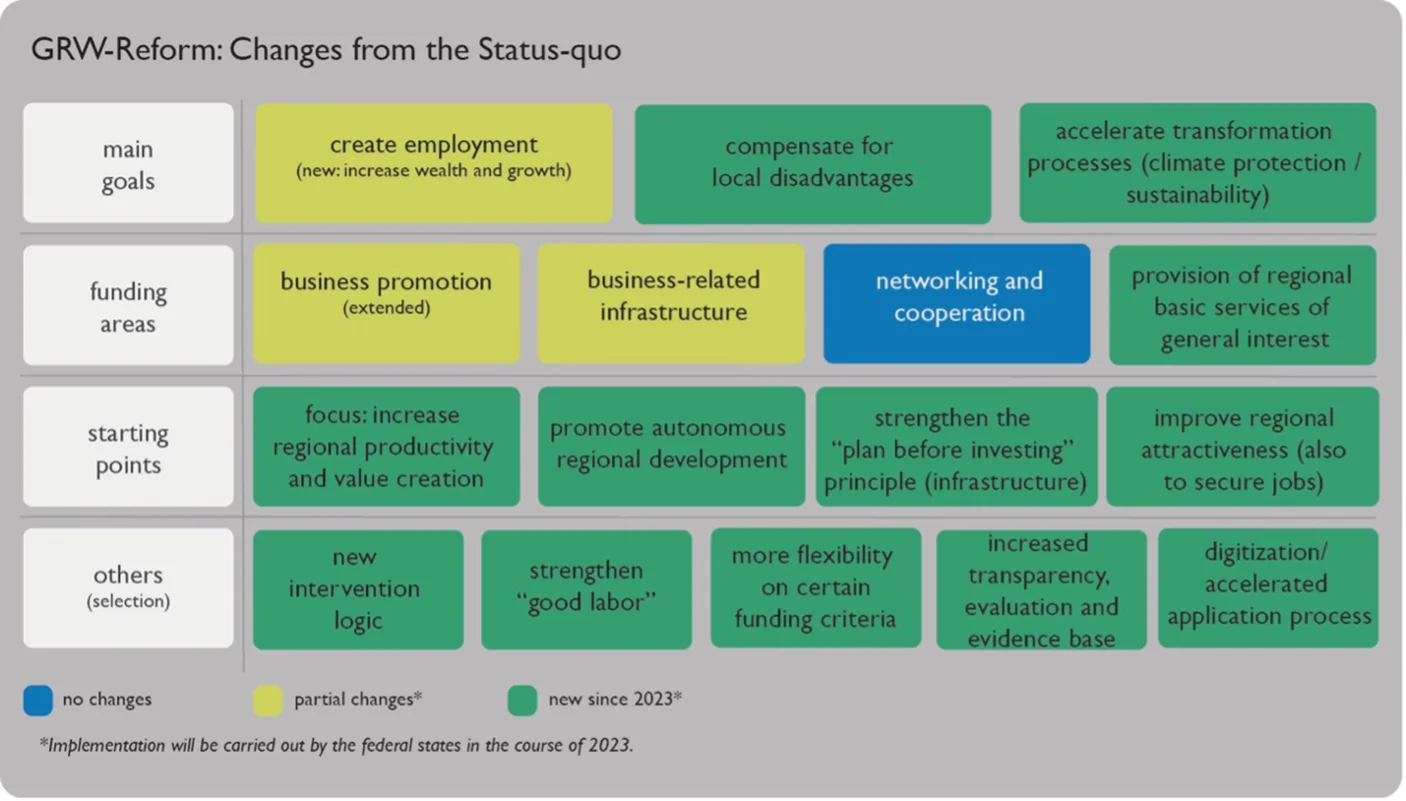
Policy changes from the status quo implemented through the recent Joint Task for the Improvement of Regional Economic Structures (GRW) reform. The table illustrates the large share of newly added aspects throughout all categories. Only the field of “networking and cooperation” remains untouched by the reform. (Source: BMWK 2022b, translated by the author)

Therefore, rather than generally questioning area-based policies, a reform of those policies was urgently needed in order to answer to the social needs that former policies targeted insufficiently. Indeed, in 2022, the German Federal Ministry for Economic Affairs and Climate Action^2^ (BMWK) reformed the GRW funding objectives and regulations with the goal of putting more focus on basic services and therefore improving living conditions in the respective areas (BMWK 2022b):

As Fig. 3 shows, the main changes, marked in green, put emphasis on factors that are not directly business-related but aim at the overarching goal of making the regions more attractive for businesses, potential employees, and residents. For the current funding period, it is planned that about half of the entire budget will be spent on those infrastructural purposes (BMWK 2022b). Whereas main goals such as “compensate for local disadvantages” are kept very general, new starting points such as “improve regional attractiveness” could indicate measures and funding for social needs. Eligible measures can be summarized as presented in slightly more detail in Table 1.

The eligible measures presented in Table 1 provide an overview of projects and interventions that could technically receive funding through the GRW scheme. The list also illustrates how aspired changes through the reform could be translated into practice. When the examples of potential measures are examined, however, it is noticeable that the focus continues to lie on business and economy-related interventions.

**Table 1:** Eligible measures of the Joint Task for the Improvement of Regional Economic Structures (GRW) funding scheme

Category	Eligible measures
Business investment support	Construction or expansion of commercial operations
	Acquisition of machinery and equipment
	Fundamental modernization or rationalization
	Innovation-oriented investments
	Investments by SMEs in structurally weak regions
Municipal economic infrastructure	Development of industrial and commercial zones
	Roads and transport links to business areas
	Water, sewage, and energy supply infrastructure
	Digital infrastructure (e.g., broadband)
	Business-related public buildings (e.g., service centers)
Tourism infrastructure (if economically justified)	Visitor centers in tourism zones
	Trails, signage, and small-scale facilities
	Environmental protection and accessibility improvements
Noninvestment measures	Business cooperation networks (clusters)
	Regional management and location marketing
	Support services for SMEs (e.g., digitization, innovation consulting)
	Coordination and qualification projects
Renewable energy and environmental measures	On-site renewable energy installations (e.g., PV for business use)
	Energy efficiency enhancements
	Exceeding of legal environmental standards
Evaluation and policy research	Monitoring and evaluation of GRW projects
	Studies on regional economic development
	Strategy development for regional policy

*SMEs* small and medium-sized enterprises, *PV* photovoltaic

Interestingly, when the previously mentioned Federal Funding System for Structural Development Regions is considered, which bundles 20 funding schemes including the GRW funding program, this network structure centered around community stakeholders, which is needed to provide (social) infrastructure and certain services, is not reflected in the list of eligible funding recipients. Like the GRW itself, almost all schemes are exclusively targeted at companies and municipalities. While it can be assumed that municipalities are the appropriate agents taking responsibility for the provision of infrastructures and services, hence ensuring a certain level of living standards, it is shown that due to limitations in both financial and human resources, this cannot be guaranteed, especially in disadvantaged areas. A critique of the funding scheme further includes the fact that local citizens have no formal say in where they subjectively feel infrastructure and services should be improved in order to increase the general satisfaction with their living environment (Küpper and Mettenberger 2020).

However, besides the largest scheme within the GFS, the GRW, other programs are less targeted at economic development and are more explicitly directed toward infrastructure and basic service improvements, with the majority of funding being targeted at disadvantaged areas. Those programs include, for example, the following (BMWK 2024a):

- Innovative Municipalities (*Kommunen innovativ*)
- Integrated Rural Development (*Integrierte ländliche Entwicklung*)
- Urban Development Funding (Städtebauförderung)
- Multigenerational house. Together - for each other (*Mehrgenerationenhaus. Miteinander* - *Füreinander*)
- Partnerships for Democracy (*Partnerschaften für Demokratie*)

Though only a comparatively small budget can be received through stakeholders other than municipalities and enterprises, the programs emphasize the fact that thanks to the reliance on voluntary work and civil society engagement, “a large leverage effect can be achieved with comparatively low funding” (BMWK 2024a, 182). Nevertheless, compared to the overall budget of the GFS, the programs listed here distribute only a rather small share of the general funding budget and an even smaller share of that funding for actions led by civil society stakeholders and citizens. They have little say in how to put the budgets to best use while seeking to accomplish better, ideally equivalent living standards in disadvantaged areas.

Recent reforms to Germany’s regional funding instruments aim to make support more flexible and better suited to specific regional needs. However, understanding the real impact of these changes requires looking beyond policy design to how they affect local residents. Regional disparities are complex, and top-down approaches may miss the nuanced realities faced by people living in targeted areas. In recent years, societal and consecutively political polarization have been attributed to growing feelings of being “left behind” in areas that have suffered from economic, structural, social, and cultural change (Rodríguez-Pose 2018; Borbáth et al. 2023; Tomaney et al. 2024). Hence, to truly grasp the consequences of these dynamics and to design more effective policies, it is essential to listen to those most directly affected.

Therefore, the next section focuses on citizens’ perspectives, based on fieldwork data from affected regions. Centering local voices offers a deeper understanding of where policies overlap with the needs of communities and uncovers gaps that policies may overlook. Incorporating citizens’ experiences is essential for the further development and improvement of regional development strategies, as both political realities and academic studies have proven the need to look beyond economic measures when assessing citizens’ well-being.

## The People: What Do Citizens in Disadvantaged Areas Perceive as Missing in Their Living Environments?

As stated above, evaluations and critics lament the persisting and even increasing level of inequality despite continuous targeting efforts by respective funding programs. Building on the previous assessment of GRW funding policies and their mixed success in addressing regional inequalities, this methodology section outlines the empirical approach to capturing residents’ perspectives. While policy evaluations focus on macro-level outcomes, this study takes a bottom-up view to understand how policies affect local communities. Combining qualitative interviews with key stakeholders and a tailored resident survey, the research highlights both tangible needs and subjective priorities. This approach provides nuanced insights into local deficits and aspirations often missed in broader evaluations in order to answer the following questions:

- What deficits do residents in structurally disadvantaged areas identify in their living environments, and which social and infrastructural factors drive their satisfaction or dissatisfaction?
- How well do existing funding schemes address identified deficits, and where do gaps remain?

The aim of this study is to explore what residents perceive as important for good living conditions and to shed light on specific deficits in regions facing particularly challenging circumstances. As an exploratory investigation, the findings are grounded in the selected case study regions and are intended to provide in-depth, context-specific insights. While the analysis may offer indications relevant to other GRW funding zone C areas, it does not claim broad generalizability beyond the cases examined.

### Case Study Locations

The empirical foundation of the second part of this research is based on surveys conducted in three case study locations in the summer and fall of 2022 and 2023. The case studies were selected based on their location in a GRW funding zone C. Whereas case study one is a village with approximately 600 inhabitants, case studies two and three represent midsized cities with approximately 31,000 and 41,000 inhabitants respectively. Whereas all region C zones targeted through the GRW funding suffer from challenging economic and infrastructural circumstances, demographic, social, and economic conditions still vary widely, in part due to very heterogeneous historical developments. Despite their location in very different parts of Germany - one in the mideast of the country and one in the far west close to the French border - both midsized cities have a history of significant population shrinkage and economic restructuring processes.

### Data Collection

To gain in-depth insights, semistructured interviews were conducted with engaged community members, local government representatives, and both professionals and volunteers from civil society organizations. Interview guides, informed by prior research and literature, were slightly tailored for public employees and volunteers, focusing on perceptions of local changes, challenges, personal involvement, and collaboration with civil society. Initial interviewees were identified through desktop research, with further participants recruited on site. Conducted in German between summer 2022 and fall 2023, interviews lasted 1–3 hours, were held in person, were transcribed automatically, and were manually coded for key themes. Findings were enriched by field observations and participation in local meetings and events. Based on these interviews, a predeveloped survey was adapted for each case study location, asking residents about satisfaction with their living environment, intentions to move, and specific needs or criticisms. Surveys were distributed both on paper and digitally via local establishments, newsletters, public spaces, and social media groups.

### Data Analysis

The qualitative interviews were recorded, transcribed, and manually coded, both deductively and inductively, for themes previously put together for the interview guides as well as for themes brought up by interviewees.

All surveys were either directly entered in the digital SurveyXact platform or manually copied and inserted from paper-based surveys. Afterward, data were cleaned, and participant entries that either did not agree to the data protection agreement or stopped answering before question four were excluded from analysis, leaving 69, 59, and 26 responses, respectively. Despite the efforts of reaching as many participants as possible, the response rates to the public surveys were low and varied across locations. Hence, we did not aim to make broad statistical generalizations. Instead, we treated the open-ended survey responses as valuable qualitative input and combined them with insights from the interviews to explore an even wider variety of insights. This way, the residents’ perspectives still played a central role in the analysis, even though the survey results cannot be considered representative. Numerical data will be partly presented in order to illustrate or underline qualitative findings of the data.

For this publication, the data entered in the text field “What do you miss in your city?” were sorted into inductively determined categoric themes. After all data entries were grouped, this led to the definition of 12 categories in total: transportation, health, youth, leisure, retail, politics, built environment, safety/accessibility, community, education, others, nothing. Data entries were edited manually to allow for frequency analysis, e.g., the data entry “Better connections in terms of public transport” was shortened to “public transport.” For reasons of accuracy, responses with a geographical focus such as “Unfortunately, the bus connection [to the city] is very poorly developed. I would be happy if this could change in the future” were changed to “public transport access to the city” and in the next step to “transportation” in order to identify repetitions and patterns. The inductively defined categories show high overlaps with categories of needs and well-being assessments discussed in academic debates, as presented in the previous section (see, e.g., Barca et al. 2012; Browne-Yung et al. 2016; Cooke et al. 2016). These scholars present well-being assessments based on categories of existing survey instruments in order to create analysis constructs to achieve results that are broadly generalizable across regions. This study, in contrast, takes a different approach by using open-text field questions and deriving categories directly from entered data. Notable overlaps with categories from larger frameworks, such as safety/accessibility, built environment, and community (Browne-Yung et al. 2016), community and politics (Barca et al. 2012), or education, community, health, and built environment (Mäki-Opas et al. 2022) illustrate similarities with scholarly considered relevant assessment categories for well-being. The widely applied WHOQOL-BREF framework (Harper et al. 1998) shows particularly strong alignment and employs subjective and objective indicators that also emerged in this study, including safety, leisure, transport, the (built) environment, and elements grouped here as “community,” such as positive feelings, social support, and social relationships.

## Findings

In order to answer the question of what residents in structurally disadvantaged areas perceive as missing or inadequate in their living environment and what contributes to their satisfaction or dissatisfaction with local living conditions, survey respondents were asked the following questions: Had they considered moving away, and if so, what made them stay? What was missing in their village/city, and what could be changed? What did they particularly like about life in their village/city, and what made it worth living in?

### Perceived Needs

**Fig. 4:**
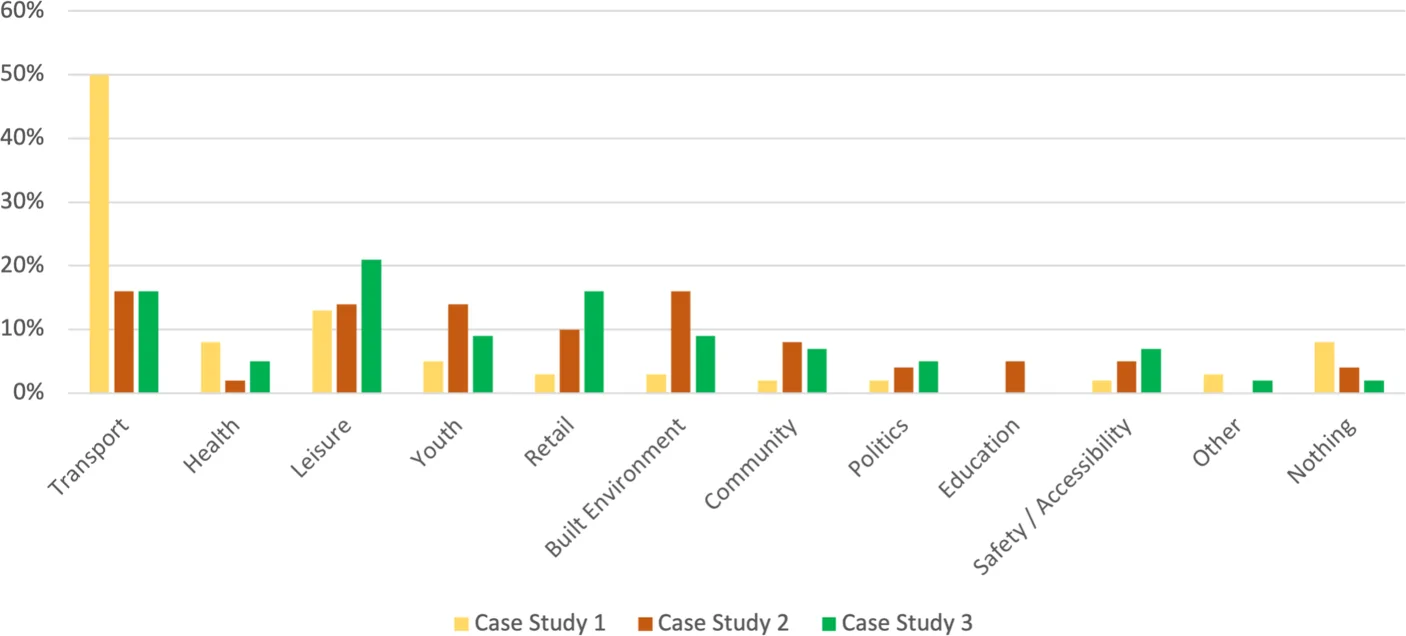
Perceived needs of residents in disadvantaged areas in Germany, survey data from 2022 and 2023

The variety of responses from all three case studies reflects the heterogeneity of demands of residents in the communities. Because the distribution is derived from qualitative data entries, participants could enter as many comments in as much detail as they liked, which were allocated into previously named categories during the analysis. The calculated and illustrated percentages (Fig. 4) reflect the number of data entries within that specific category for each case study location, respectively.

Figure 4 (4) shows how priorities vary between the three cases. In the first case study, the rural community, more than 50% of all data entries were related to transportation. Among those, most comments criticized the poor access to public transportation within the village, most of which specifically referred to the bus connection to the next larger city, which, as the state capital, serves as an infrastructural and administrative center for the surrounding communities. Other comments within the transport category included the wish for a lower speed limit on the main road and better-quality sidewalks. The second most frequently cited category was “leisure,” which in most cases was noted with considerably less urgency and referred, for example, to the wish to have a lake close by.

For case study 2, a midsized city affected by significant population loss, demographic shifts, and economic restructuring, residents’ needs were varied but strongly focused on the built environment, transportation, and community amenities. Many respondents highlighted the abundance of abandoned buildings, lack of renovations, and poor cleanliness in the city center, all contributing to its deteriorating appearance. The limited availability of attractive shops, dining options, and youth activities was frequently linked to demographic change. As one respondent observed, “There is generally a lack of appeal for young people to move here. A university could possibly be a good way to attract more people. You just feel like you are living in a big retirement home.”

In a separate survey question, nearly one in four respondents across the two city case studies reported having considered moving away for job-related reasons. Yet when asked openly about their needs or priorities for change, employment was rarely mentioned. Instead, concerns focused more on the decline of local amenities and quality-of-life factors. This pattern is echoed in case study 3, in which participants criticized the lack of leisure, retail, and public transportation options. Similar to case study 2, this city was once a thriving economic and cultural hub with a larger population and vibrant social life. The contrast with current infrastructure and services left many long-term residents feeling frustrated and nostalgic. As one respondent summarized, what is missing are “financial resources to revitalize the city center, to stimulate the economy, especially in terms of retail, and to lure people back to the center. Activities for children have also become very rare, as have opportunities for young adults to go out, but events like the pub tour show there is no lack of interest.” This highlights the surprising dynamic that, despite potential economic concerns, residents primarily emphasize improvements to local living conditions over economic issues. This result is underlined when comparing the inductively coded categories with the categories applied in studies of broader well-being frameworks. Public transportation, leisure options, youth services, and retail offers are largely absent from most established well-being frameworks. Only a few, such as the WHOQOL-BREF, include select aspects such as transport and leisure, highlighting how these everyday features remain underrepresented in many broader assessments. It appears that these factors are deeply rooted in the realities of structurally disadvantaged environments, reflecting both the erosion of local services and the decline of small, individual businesses. Interviews emphasize that their absence leaves lasting negative impressions on local residents’ daily lives and profoundly shapes their perception of their living environment such that it tends to feel less vibrant and urban than they recall from the past.

### Perception, Pessimism, and Self-efficacy

A category that played no role in the rural case study but seemed rather relevant in both case studies 2 and 3 is the mentioned pessimism among local residents, which was categorized as “community.” Together with the perceived lack of meeting spaces - especially for youth - this seems to determine much of the atmosphere among the local populations, even though the extent of this differs between the two cases. While this was already indicated in the survey responses, participants in the more elaborate semistructured interviews similarly highlighted the importance of the community’s mentality. In case study 1, there was general praise for the development of the village and the community’s engagement in order to provide services and infrastructure despite the remote location of the village. This is also reflected in the comments on what is missing in the village, namely public transportation, something that is more challenging to organize and provide on a community level. Nevertheless, while at the time of data collection in the summer of 2022 there was only the wish for better transportation and the distant plan of some of the interviewed engaged citizens of the village to organize a community bus, the village community again has managed to turn this plan into action. By 2024, the citizens had managed to turn a former van of their volunteer fire department into a citizens’ bus. The bus operates on demand with volunteer drivers from the village community and can be used by any member of the village association. Additionally, by 2025, the engaged citizens had managed for the village to become part of a pilot project of the regional public on-demand bus system, using an electric van with remunerated volunteer drivers to connect the village to public transport in neighboring communities.

In case study 2, a rather high level of dissatisfaction with political and administrative leadership was conveyed, which was less targeted at individual personnel itself but rather at the political administrative system. Engaged citizens themselves felt not seen and politically integrated enough considering their efforts for the public good. Weekly demonstrations within the city were, according to the mayor of the city, also less targeted at him as a person but rather at the national government and policies that seem to disregard the challenges on the local level. In case study 3, interestingly, the survey participants mentioning the prevailing pessimism within the city did so rather critically: “There is nothing missing except for greater self-confidence among the residents. A less pessimistic view of one’s own city could help a lot!” Despite the challenging economic situation of the city paired with sociodemographic challenges, several respondents emphasized the need to tackle those challenges collectively, a spirit that is also confirmed by the coordinators of citizen initiatives, who rely on a stable and considerably large network of volunteers who contribute to the provision of (free) social services and offers to the public.

### Political Leadership

During the interviews with the political leadership in the two midsized cities, it became clear that the approaches to tackling local issues were quite different. Whereas both case study cities 2 and 3 have suffered from the consequences of demographic change and shrinkage, further contributing to economic hardships, both cities employ rather different tactics in addressing these challenges. Though citizens in both cities emphasized the need for internal urban development measures that increase the quality of life for the local communities, this is not the main strategy of both political leadership schemes. In case study 2, the mayor emphasized the need to attract newcomers and tourists to revive the city; hence, a majority of investments are targeted at measures that increase the attractiveness of the city for visitors. Large parts of the investments needed for these measures will come from the federal Structural Strengthening Act for Coal Regions,^3^ for which the city qualifies. Some of these large investments in upcoming years will create or revive tourist hot spots (entertainment and cultural institutions), which are hoped to attract enough outsiders to then create more business for local retail and gastronomy - two factors frequently mentioned by survey respondents as things they particularly miss in their city.

In case study 3, the mayor answered the question about measures to put a halt to the vicious cycle of decline rather differently. Rather than individual lighthouse projects, he emphasized the need for financial redistributive mechanisms, highlighting the fact that due to a high share of secondary migration in recent years and the generally large share of welfare recipients within the city, the financial budget was kept at a minimum that does not allow for larger investments. Second, he emphasized the need to provide support to social and civil society organizations in the city, which through their volunteer networks cushion what the city cannot provide. Therefore, one of his priorities is the close collaboration with institutions like the senior citizen center, the youth education program, or the neighborhood community centers, which are supported through the provision of full-time staff.

These varying priorities of the political leadership in the different locations highlight the vast differences in circumstances under which different civil society organizations work. Whereas interviews in all three case studies reveal the high level of engagement and motivation of individuals to contribute to better living standards in their respective home community, the obstacles they face and the support they receive in carrying out their services differ significantly. In case study 1, volunteers ensure the running of the local village store and the village library, offer almost daily sports club activities for different target groups, maintain public meeting spaces such as a “bee meadow,” and, depending on demand, e.g., during the COVID-19 lockdowns, offer delivery services for older or handicapped residents of the village. Large investments, such as the expansion and furnishing of the village store, were financed through external grants that members of the community applied for in collaboration with the (due to the small size of the community, unpaid volunteer) mayor.

In case study 2, civil society organizations operate entirely independently from the local administration, except for informal support through individual members of the administration, who, for instance, assist with the acquisition of spaces. All financial and administrative support is individually recruited, and the majority of initiatives in this city benefitted from a large national grant that one of the organizations received through winning a competitive selection process.

In case study 3, as was emphasized through the mayor’s priorities in strengthening civil society organizations that take over important social services in the city, most of the identified and interviewed organizations receive support in the form of paid staff and premises. Hence, volunteering residents can fully focus on the provision of activities and services.

## Discussion: Assessing the Match Between Funding Allocations and Population-Identified Needs in Disadvantaged Communities

This latter dynamic deserves more attention in detail. There is a strong dependence on civil society engagement in order to provide living standards acceptable to residents in disadvantaged areas. Nevertheless, the ways in which local administrations and institutions support these vary widely. Other studies have focused in more detail on the forms of collaboration between government institutions and civil society stakeholders. Instead, this discussion will focus on the way in which main policy funding schemes recognize and address this importance of civil society engagement after having shed light on what the general local population perceives as relevant and/or missing in their living environment.

As illustrated in Fig. 2, the two most frequently mentioned fields of critique of the citizens are transport and leisure. The different geographical units do play a role in the emphasis that residents seem to put on different aspects of their living environment. It appears that residents in the village are aware that limited shopping and infrastructural opportunities can be expected due to the small size of the village. This is supported by the fact that five out of eight responses categorized as “leisure” in the village refer to the fact that a “lake nearby” would be nice - something that the residents do not expect to happen or “need” but mention as something they would like. In contrast, the fact that the large majority of responses in the village refer to the transport situation is due to the poor connection between the village and the next larger city. Although people see this as an infrastructural task for public institutions, interviews revealed that similar to the self-responsible establishment of retail and sports opportunities, some engaged members of the community had already started brainstorming how to fill the gap left by poor regular public transport through a community-organized transportation service, which they managed to accomplish within less than 2 years. The fact that this service, which is commonly perceived to lie within the responsibility of public institutions, is in the hands of the engaged community highlights the issues of the GRW funding scheme:

Using public transport access as a common example, Table 2 highlights the challenges of applying GRW funding to projects that primarily improve residents’ living conditions in disadvantaged areas. While better transport links could significantly enhance access to jobs and services in nearby cities, the scheme restricts funding to projects with a clear and direct business link. Long-term costs, such as operations, are especially difficult for already underfunded communities to cover, illustrating how the scheme falls short in addressing broader living and working conditions.

**Table 2:** Eligibility of bus infrastructure and operations under the Joint Task for the Improvement of Regional Economic Structures (GRW)

Needed item/service	GRW eligible?	Notes
New bus stop	Possibly	Must be tied to business infrastructure (e.g., an industrial zone)
Running costs of the bus line	Not eligible	Operating expenditures are excluded under GRW rules

In the mid-sized cities of case study 2 and 3, after transportation, leisure was the second most frequently mentioned category. In contrast to the village, where the wish for a lake does not contribute to dissatisfaction with the living environment, the demand for more leisure activities in the midsized cities stems from severe frustration about the lack of meeting spaces and activities. Survey responses and interviews reveal the importance of the historical development of both examined cities, which used to be vibrant urban centers several decades ago - a time that many of the remaining residents still remember - before economic and demographic decline caused the shutdown of most gastronomic establishments and meeting spaces. The same goes for the retail offers, which were the third most frequently mentioned category. Residents in the declining cities are constantly exposed to barricaded shop fronts, which, similar to the lack of gastronomy, serve as visual reminders of the perceived decline of their hometowns.

The GRW reform acknowledges the fact that the former exclusive focus on economic measures, mostly related to the employment and business opportunities within the regions, did not have sufficient effects on the perceived quality of life for people living in disadvantaged areas. Hence, as illustrated in Fig. 3, a new funding area named “provision of regional basic services of general interest” (*Regionale Daseinsvorsorge*) was strengthened through the most recent reform of the program. Along with this new focus, the previous funding criterion of businesses having to focus on supraregional business activities was abolished in order to also support businesses with local and regional focus.

Parallel to the reform of the largest structural funding program, the whole federal funding system has been redesigned and unified. The new system consists of more than 20 programs that are targeted at the establishment of equivalent living conditions throughout Germany. Due to the federal system of Germany, funds are mostly channeled from the federal state toward the substates (*Länder*) and municipalities. Whereas the GFS is targeted at structurally disadvantaged areas, other areas can apply for and receive funding as well, as long as the majority of funds are targeted at disadvantaged areas. Although this is thought to support preventive measures in order to avoid municipalities from qualifying as disadvantaged in the future, this does channel large sums of funding away from those areas most in need.

Further, the new GFS system was supposed to simplify and unify the previous funding landscape, which proves challenging when looking at overlaps and distinctions between the different programs. Illustrated by the previous discussion on what equivalent living standards entail and what can be considered “basic” services and infrastructure, the different programs set different priorities. Although this diversification certainly makes sense in order to reach as many target areas and stakeholders as possible, it does make the funding landscape challenging to navigate. Not only do eligible funding recipients differ between each program, but so do the agencies that are implementing the programs in the different states of Germany.

Research and policies acknowledge the fact that civil society organizations and stakeholders play an increasingly important part in the improvement of local living standards, yet few programs are targeted at their support, and even most recent funds, such as the Structural Strengthening Act for Coal Regions and its second-pillar programs, do not allow for citizen organizations to be primary grant recipients. Programs allowing for applications from civil society stakeholders are mostly project-related and are targeted at benefits in kind and networking expenditures, relying on citizens to commit their free time. This does not only increase the challenge of finding enough volunteers in many places but also shifts responsibilities for local infrastructures to citizens, who are burdened with the administrative tasks of their engagement in addition to employment, education, and family duties.

Though the places chosen for surveys and interviews all have a considerable share of volunteer-based activities and services, the impact these have differs significantly between the three case study locations. Whereas in the village (case study 1) the strong local community has a wide-reaching impact whose efforts and offers reach a large share of the local population, experiences from the midsized cities show that in a city, especially one in which shrinkage and abandoned buildings create a dispersed urban structure, the effect of individual initiatives that are exclusively based on volunteers is rather limited. What people there mentioned most frequently as missing mostly refers to a more vibrant urban center—which they mentioned in the form of retail choices, more modern gastronomic offers, more cleanliness, and upgrades in the built environment, which mostly point to decaying buildings and abandoned shop fronts. For volunteers and civil society organizations to address these perceived needs, the multifold administrative and financial burdens that come with such measures make such changes very challenging and difficult to implement. Instead, urban development policies and municipal stakeholders should be supported in a way that allows them to prioritize such measures. The fact that most funding schemes are targeted at municipal institutions as funding recipients seems to acknowledge this fact but disregards the persisting lack of human resources prevalent in most municipalities in disadvantaged areas. This is also seen as one of the reasons why many funding pots are not fully utilized. Additionally, the persisting bottleneck of human and financial resources in those municipalities further impedes any collaborative process with civil society in order to prioritize measures and establish potential cooperation with other community stakeholders, as those processes tend to be rather resource intense.

Further data emphasize the urgency to target more funding and resources specifically at civil society stakeholders: When matching the perceived needs of the local population with current stakeholders providing exactly those types of services, it becomes clear that a large share of them are already provided through civil society initiatives and independent projects and organizations. Speaking with the different initiative leaderships, however, revealed the heterogeneity of administrative and financial support organizations receive in different municipalities. Local key organizations in case study 3 receive reliable, permanent support through full-time staff being paid by the local administration, while most actors in case study 2 are dependent on externally acquired grants, which again create a hierarchy and dependency among the different civil society organizations. In case study 1, general running costs are very low due to community premises within the central village, and larger costs for construction works and purchases had to be covered through external grants acquired by engaged members of the community, which include the administrative leadership represented by the mayor.^4^

Case studies 1 and 2 particularly highlight the challenge of creating reliable structures of civil society institutions, as their communities’ capability to acquire funding is highly dependent on the skills and capacities of individual members of the community.

Especially in light of resource restraints in public administrations, there is a great lack of supporting structures that could help civil society stakeholders in writing grant proposals and applications or with the general administration of funding schemes and legal procedures.

Looking at the changes following the GRW reform, the need for better provision of services and attractiveness of local living conditions was taken into account when reforming the new directive. Nevertheless, the reality of funding eligibility and the bureaucratic obstacles for both administrations and external parties for funding applications were insufficiently taken into consideration. Especially considering the high relevance of civil society stakeholders, accommodation of their needs was hardly sufficiently recognized in the reform, and the stakeholders continue to be dependent on individual funding applications, high use of personal resources, and the goodwill of a collaborative local administration in order to receive support.

## Conclusion

This research article looked at the main public funding tools aimed at leveling living standards across Germany, with a focus on how well the funding schemes support community-centered investments. In the second part, fieldwork data from three case-study sites in disadvantaged areas help identify local needs and priorities for their living environment. Finally, both the policy review and the field data tackle the following questions:

- What deficits do residents in structurally disadvantaged areas identify in their living environment, and which social and infrastructural factors drive their satisfaction or dissatisfaction?
- How well do existing funding schemes address identified deficits, and where do gaps remain?

To respond to these questions, the answers draw on survey responses, interviews, field observations, and a policy assessment of the largest funding schemes in Germany targeted at alleviating regional inequality and improving local living standards. In response to the first research questions, deficits as perceived by residents in disadvantaged areas were the main focus, as well as factors that drive their satisfaction or dissatisfaction with living in the respective localities.

Because the cities in case studies 2 and 3 are especially suffering from the consequences of demographic change and economic restructurings in the past, the question on considerations of moving away was particularly relevant. Based on academic debates and policy priorities, the expectation was that this would open up conversations on economic and employment challenges, but while one in four residents indicated that they had considered moving for job-related reasons in the past, the deficits impacting their perception of their living environment were almost exclusively focused on social and infrastructural aspects. The most frequently cited shortcomings were gaps in local transportation, a lack of leisure activities and meeting spaces, and declining gastronomic and retail options.

This prioritization of community-centered needs over purely economic issues feeds into a general mood of pessimism that many residents described, especially in the two city case studies, in which decline has been visible in the form of abandoned buildings, barricaded shop fronts, and diminished social offers for decades. Even where citizen organizations receive strong municipal support and the local administration prioritizes local measurements directly targeted at better living standards for local residents, participants mentioned that “nothing is missing except greater self-confidence among the residents,” suggesting that it is community cohesion and local confidence that are missing to transform the city’s atmosphere amid economic and demographic challenges.

This potentially almost positive outlook on required changes in the city is not shared by the participants in the other urban case study, where local residents and citizen organizations do not seem to feel seen by investment priorities set by the local government. There, citizen organizations perceive their own efforts as undervalued by the local governments, a feeling that, besides a perceived lack of political integration, is also traced to a lack of structural and financial support.

Satisfaction with local living conditions hinges on the availability of services and spaces that foster social life and connectivity. Yet initiatives to improve these aspects rely heavily on unpaid volunteer work, which limits sustainability and reach.

Responding to the second research question, the article assessed the main public funding instruments aimed at achieving equivalent living standards throughout Germany to see how well they meet the needs of local communities in disadvantaged areas. The examination of the GRW reform shows a shift of the largest area-based funding scheme in Germany. As the primary tool for targeting regional inequality, it has moved away from purely economic criteria to include measures targeted at improving living conditions. The review also revealed, however, that despite this shift, it remains challenging to use nationally allocated funds for the purposes most relevant to local residents in their areas. Many of their needs and demands are instead taken up by civil society stakeholders. The extent to which they receive administrative or financial support, however, remains entirely at the local administration’s discretion, often leading to the organizations to need external grant acquisition and extensive unpaid efforts of engaged community members.

Bureaucratic obstacles impede acquisition even where funding is technically available, as many disadvantaged municipalities lack the personnel and financial stability to apply for funds and implement them effectively. This structural constraint reduces the policy’s responsiveness to local needs.

We conclude that there has been progress by recognizing the importance of local living standards rather than focusing almost exclusively on economic measures. The reform has significantly improved Germany’s largest scheme for equal living standards, but the points listed above show persistent shortcomings despite the positive direction of implemented changes. Future reforms need to acknowledge that public administrations alone - especially in times of staff shortages and tight budgets - cannot be the sole drivers of improved living conditions in disadvantaged areas. The perceived needs and demands voiced by residents highlight the importance of integrating nongovernmental stakeholders into local institutional structures. A stronger focus on citizen-led initiatives and social infrastructure is not only necessary but feasible within the existing European Union state aid and competition law framework. Only through such institutional integration, backed by appropriate policy and financial support, can civil society actors become a sustainable pillar in strengthening living standards in disadvantaged regions.

## Supplementary Information

The Supplementary Information includes the community survey used in City 1, adapted to preserve anonymity.

## Data Availability

The data supporting the findings of this study are available from the author upon reasonable request. However, access is restricted in order to protect the privacy and confidentiality of the participants.

## Footnotes

1 Article 72(2) of the German Basic Law, which calls for the establishment of equivalent living conditions, provides the constitutional basis for the GRW (as do other provisions, including Section 2(1) of the Regional Planning Act). (Source: Framework for the Coordination of the Joint Federal/Länder Task for the Improvement of Regional Economic Structures [GRW] as of 1 January 2022).

2 During the legislative period from 2021 to 2024, the BMWK was headed by Robert Habeck, minister of the Green Party (*Bündnis 90 die Grünen*). Following the snap elections in 2025, the ministry was renamed the Federal Ministry for Economic Affairs and Energy (BMWE) and came under the leadership of Katherina Reiche, a member of the Christian Democratic Union.

3 The Structural Strengthening Act for Coal Regions (*Strukturstärkungsgesetz Kohleregionen*) commits up to 40 € billion in federal funding by 2038 to support structural and economic development of former lignite and coal mining areas. Second-pillar programs of the fund are likewise targeted at federal states and public institutions as primary grant recipients and can only indirectly fund civil society stakeholders in the form of project partners.

4 Volunteer public officials in that state receive an expense allowance and are typically supplementing reimbursement with their primary employment.
