# Supplementary material for: Challenging Growing Regional Inequality: Area-Based Policies in Germany as a Path to Equivalent Living Standards?
Source: Polit Vierteljahresschr. 2025 Oct 15;67(3):477–504. doi: 10.1007/s11615-025-00636-4 (PMC13503465; doi:10.1007/s11615-025-00636-4)
Supplement: Supplementary file 1 [file 11615_2025_636_MOESM1_ESM.pdf]

Supplementary material

for

Challenging Growing Regional Inequality: Area-Based Policies in Germany as a Path to Equivalent Living Standards?

Naomi Alcaide Manthey[1]

Supplementary material: Anonymized community survey as used in City 1 during the field work in the summer of 2022.

[1] **Author affiliation**: University of Copenhagen, Department of Geosciences and Natural Resource Management, Copenhagen, Denmark
E-mail: nalm@ign.ku.dk, **ORCID:** 0000-0001-9288-8155

[City 1]: Citizen Engagement in Disadvantaged Areas

Consent to data processing

Before you can start with the questionnaire, we ask you to read the information on the processing of your personal data:

Please click here: <u>Information sheet on data processing</u>

❑ I hereby confirm that I have read the document "Consent to the processing of your personal data by a research project" and agree to the terms and conditions.

Your life in [City 1]

For how many years have you lived in [City 1]?

________ years

What part of [City 1] do you live in ?

❍ Zentrum / Center

❍ ...

❍ Outside of the city within the region

❍ I live elsewhere

Are you currently or have you in the past considered moving away from [City 1] ?

❍ No, never

❍ Yes, for family-related reasons

❍ Yes, for job-related reasons

❍ Yes, for health-related reasons

❍ Other reasons: ____________________________

If you answered the previous question with « Yes », please briefly describe what made you stay in [City 1]:

What is missing in [City 1]? What could be changed?

What do you particularly like about life in [City 1]? What makes the city worth living in?

Generally, how content are you with your home town?

❍ very good

❍ good

❍ so so

❍ not so much

❍ not at all

❍ I don't know

Please indicate how well you personally find your access to the following infrastructural facilities in [City 1]:

	Very good	good	mixed	Not so well	bad	Don't know
Public transport	❍	❍	❍	❍	❍	❍

Long-distance traffic	❍	❍	❍	❍	❍	❍
Grocery stores	❍	❍	❍	❍	❍	❍
Drug stores	❍	❍	❍	❍	❍	❍
Medical care	❍	❍	❍	❍	❍	❍
Cultural institutions	❍	❍	❍	❍	❍	❍
Religious institutions	❍	❍	❍	❍	❍	❍
Job market	❍	❍	❍	❍	❍	❍
Educational facilities	❍	❍	❍	❍	❍	❍
Child care	❍	❍	❍	❍	❍	❍
Gastronomy	❍	❍	❍	❍	❍	❍
Green spaces	❍	❍	❍	❍	❍	❍
Meeting places	❍	❍	❍	❍	❍	❍
Sports and leisure facilities	❍	❍	❍	❍	❍	❍

Engagement and volunteering

Are you engaged in volunteer work?

❍ Yes, in [City 1]

❍ Yes, outside of [City 1]

❍ No

❍ I am not sure

In case you answered the previous question with « Yes », please briefly describe what motivates you to volunteer.

In case you answered the previous question with « No », please briefly describe why you (currently) do not engage in volunteering.

Independent of whether you are currently involved in volunteering or not, what do you deem the most motivational factor for people to get engaged?

❍ Financial rewards / compensation

❍ Appreciation in the form of coupons

❍ Awards

❍ Social exchange and friendships

❍ Training opportunities

❍ Societal impact

❍ Change from everyday life

❍ Something else: _____

Which institutions / initiatives / projects /associations do you feel contribute positively to life in [City 1]?

(You are welcome to explain why you consider the different stakeholders to have a positive impact).

Society & Community

Do you have friends or relatives with whom you are in close contact ?

❍ Yes, several

❍ Rather yes

❍ Rather no

❍ No, none

How do you participate in public life in [City 1]?

(multiple answers possible)

❍ I work in [City 1]

❍ I am actively involved in an association or regular activities

❍ I only participate in occasional events or festivities

❍ I maintain many personal acquaintances in [City 1]

❍ I volunteer in [City 1]

❍ I use offers, services and institutions in the city

❍ I have children who are involved in institutions / activities in [City 1]

❍ I rarely participate in public life

❍ Others: _____

Societal participation: How well do you feel integrated into the urban community?

❍ very good

❍ good

❍ so so

❍ not so well

❍ not at all

Who do you get in touch with when you have a concern related to life in [City 1]?

(multiple answers possible)

❍ City administration

❍ Association or initiative

❍ Church community

❍ Friends or relatives

❍ Engaged individuals

❍ Doesn’t affect me (yet)

❍ I wouldn’t know who to contact

❍ Others: ______

Political representation : How well do you feel politically represented through the following institutions ?

	very good	good	so so	not so well	not at all	I don’t know
City administration	❍	❍	❍	❍	❍	❍
District council	❍	❍	❍	❍	❍	❍
State government	❍	❍	❍	❍	❍	❍
National government	❍	❍	❍	❍	❍	❍

Politics and participation : Which of the following statements do you agree with?

(multiple answers possible)

❍ The population receives good information on political topics and decisions

❍ I see my interests well represented in the city

❍ The population can share their opinion on a lot of political topics

❍ The population’s opinion is actively sought after within the process of important political decisions

❍ The population can actively participate in important political decision-making processes

❍ I don’t know, whether there is a chance to participate, but I would like to have a say

❍ I prefer to stay out of political discussions and decisions

❍ I don’t participate because my opinion will not be heard

Personal information

Gender:

❍ Female

❍ Male

❍ Divers

How old are you ?

_______ years

How many people live in your household?

Adults: _______

Children under the age of 18 : _______

What is your employment status ?

(multiple answers possible)

❍ In training

❍ Marginal employment («geringfügig beschäftigt ») / 450 Euro-job

❍ Employed (full-time)

❍ Employed (part-time)

❍ Self-employed

❍ Retired

❍ Unemployed

If you are employed, please indicate where your work place is located:

❍ I work in [City 1]

❍ I work outside of [City 1] in a city

❍ I work outside of [City 1] in a rural area

❍ Other: _____

Do you work from home (home-office) ?

(If you respond with « yes » please indicate the number of days per week that you work from home)

❍ No, never

❍ No, only during Covid-19 pandemic regulations

❍ Yes, regularly since the Covid-19 pandemic for these many days per week : _____

❍ Yes, always on this many days per week: _____

What is your average gross household income (in Euro)?

Providing this data is voluntary. Gross household income refers to the sum of all gross incomes of members of the household : Income from employment, pension payments, social welfare benefits, rental income, etc. A rough estimate is sufficient.

________________________________ Euro

Thank you for your participation. If you have further comments, please leave them here:

As soon as you click « Finish » your responses will be submitted and the questionnaire will be closed.

Thank you !
